# Associations between *XPD* Asp312Asn Polymorphism and Risk of Head and Neck Cancer: A Meta-Analysis Based on 7,122 Subjects

**DOI:** 10.1371/journal.pone.0035220

**Published:** 2012-04-20

**Authors:** Yuan Yuan Hu, Hua Yuan, Guang Bing Jiang, Ning Chen, Li Wen, Wei Dong Leng, Xian Tao Zeng, Yu Ming Niu

**Affiliations:** 1 Department of Stomatology, Taihe Hospital, Hubei University of Medicine, Shiyan, People's Republic of China; 2 Institute of Dental Research, Nanjing Medical University, Nanjing, People's Republic of China; 3 Department of Radiology, Taihe Hospital, Hubei University of Medicine, Shiyan, People's Republic of China; 4 Department of Dermatology, Taihe Hospital, Hubei University of Medicine, Shiyan, People's Republic of China; Dartmouth College, United States of America

## Abstract

**Background:**

To investigate the association between *XPD* Asp312Asn polymorphism and head and neck cancer risk through this meta-analysis.

**Methods:**

We performed a meta-analysis of 9 published case-control studies including 2,670 patients with head and neck cancer and 4,452 controls. An odds ratio (OR) with a 95% confidence interval (CI) was applied to assess the association between *XPD* Asp312Asn polymorphism and head and neck cancer risk.

**Results:**

Overall, no significant association between *XPD* Asp312Asn polymorphism and head and neck cancer risk was found in this meta-analysis (Asn/Asn vs. Asp/Asp: OR = 0.95, 95%CI = 0.80–1.13, *P* = 0.550, *P*_heterogeneity_ = 0.126; Asp/Asn vs. Asp/Asp: OR = 1.11, 95%CI = 0.99–1.24, *P* = 0.065, *P*_heterogeneity_ = 0.663; Asn/Asn+Asp/Asn vs. Asp/Asp: OR = 1.07, 95%CI = 0.97–1.19, *P* = 0.189, *P*_heterogeneity_ = 0.627; Asn/Asn vs. Asp/Asp+Asp/Asn: OR = 0.87, 95%CI = 0.68–1.10, *P* = 0.243, *P*_heterogeneity_ = 0.089). In the subgroup analysis by HWE, ethnicity, and study design, there was still no significant association detected in all genetic models.

**Conclusions:**

This meta-analysis demonstrates that *XPD* Asp312Asn polymorphism may not be a risk factor for developing head and neck cancer.

## Introduction

Head and neck cancers (HNC) constitute about 5% of all cancers recorded in the US, and the incidence is increasing in most developed and developing countries. These cancers have been estimated to be about six times more common among smokers than non-smokers and are most common in males over 50 years old [1], [2], which increases to about 15 times if the smokers are also heavy drinkers [3], [4]. Although many measures had been done to improve the diagnosis and treatments, the prognosis was still poor.

Many environmental factors, such as radiation, diet, smoking, and endogenous or exogenous estrogens, are associated with DNA damage. Unrepaired or misrepaired DNA results in gene mutations, chromosomal alterations, and genomic instability. Several studies have suggested that genes involved in DNA repair play a crucial role in protecting against mutations. Patients with certain cancers have reduced capacities for DNA repair. Similarly, the enzymes of the nucleotide excision repair (NER) pathway have been implicated in cancer. Associations between polymorphisms in several DNA repair genes and the risks of several types of cancer have been extensively examined. Many epidemiologic cancer studies have focused on single nucleotide polymorphisms (SNPs) in genes in the NER pathway such as *XPD*, *ERCC1*, and *XPC* [5]. The XPD protein is a DNA helicase and is an essential part of the TFIIH transcription factor complex. Some studies have suggested that *XPD* polymorphisms may be associated with reduced DNA repair because of a possible reduction in helicase activity [6], [7]. One of the common *XPD* polymorphisms in the coding regions is Asp312Asn in exon 10. The functional significance is not yet completely clear, although the amino acid mutations in exon 10 give rise to a loss of an acidic residue and a complete change in the electronic configuration of the amino acid [8], [9].

The first study on the relationship between HNC and *XPD* Asp312Asn polymorphism was conducted by Sturgis et al. [10]. They found a borderline significant association between *XPD* Asp312Asn polymorphism and HNC. Since then, a lot of studies have confirmed or refuted this finding [11]–[18]. In 2010, a recent meta-analysis was conducted by Flores-Obando et al. [19] demonstrated that increased HNC risk is associated with *XPD* Asp312Asn polymorphism. Worthy of note, that meta-analysis included five studies were conducted in Caucasian populations and one in an Asian population [10]–[15]. Today, nine case-control studies on *XPD* Asp312Asn polymorphism and HNC risk have been published. A comprehensive meta-analysis is needed to provide an updated approach on the overall relationship. Subgroup analyses were also performed on Caucasian and Asian populations to investigate ethnicity-specific effects.

## Methods

### Search strategy

The PubMed database was searched with terms “head and neck cancer", “oral cancer", “oropharyngeal cancer", “laryngeal cancer", “pharyngeal cancer", “XPD", “excision repair cross-complementing group 2", “polymorphism", and the combined phrases for all genetic studies on the relationship between *XPD* polymorphism and HNC risk from 2000, when the first study of the association between *XPD* Asp312Asn polymorphism and HNC risk was reported, to October 2011. We also used the “Related Articles" option in PubMed to identify additional studies on the same topic. Reference lists in retrieved articles were also screened for. All selected studies complied with the following three criteria: (a) case–control study on the *XPD* Asp312Asn polymorphism and HNC risk; (b) sufficient published data for estimating the odds ratio (OR) with 95% confidence interval (CI); (c) For multiple publications reporting on the same data or overlapping data, the largest or most recent publication was selected [20].

### Data extraction

Two investigators (Hu and Yuan) independently extracted the following data from each included publication: the first author's name, publication data, sources of controls, racial descent of the study population (categorized as either Asian or Caucasian), genotyping method, number of cases, cases and controls with different genotypes, and Hardy-Weinberg equilibrium(HWE).

### Statistical analysis

Crude ORs with 95% CIs were computed to assess the strength of the correlation between the *XPD* Asp312Asn polymorphism and HNC risk. The pooled ORs were performed for codominant model (Asn/Asn vs. Asp/Asp,Asp/Asn vs. Asp/Asp), dominant model (Asn/Asn+Asp/Asn vs. Asp/Asp), and recessive model (Asn/Asn vs. Asp/Asp+Asp/Asn), respectively. In the subgroup analysis, statistical analysis was conducted on Asians and Caucasians. Heterogeneity assumption was assessed by the chi-square based Q-test [21]. The pooled OR estimation of each study was calculated by the fixed-effects model (the Mantel–Haenszel method) when *P*>0.10. Otherwise, the random-effects model (the DerSimonian and Laird method) was used [22]. The potential publication bias was estimated by the modified Egger's linear regression test, which proposed by Harbord et al. [23]. Statistical analysis was performed using STATA version 11.0 (Stata Corporation, College Station, TX, USA) and Review Manage (v.4.2; Oxford, England), using two-sided P-values, with *P*<0.05 considered statistically significant.

## Results

### Study characteristic

**Figure 1:**
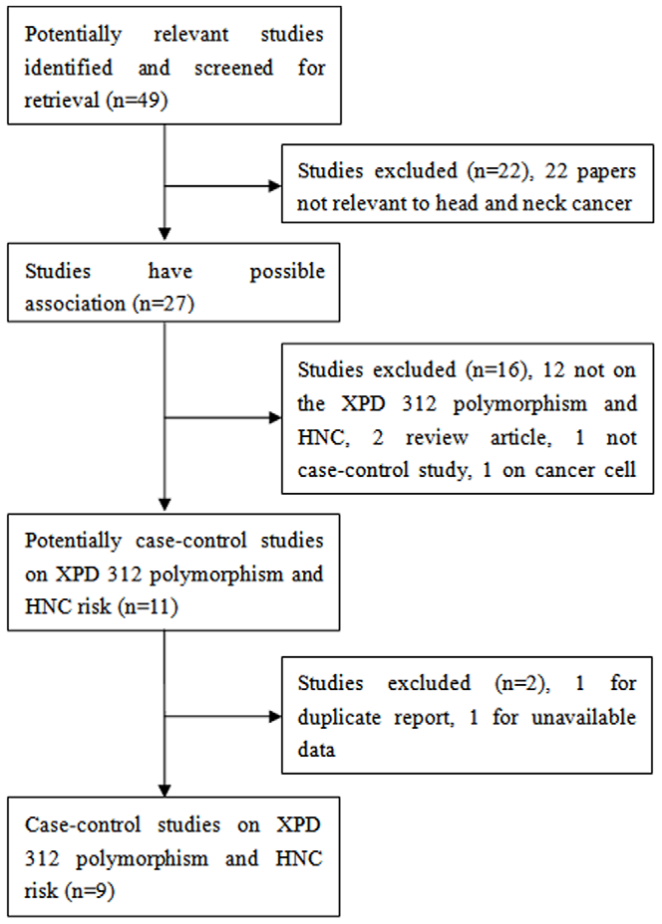
Flow diagram of the study selection process.

This meta-analysis is guided by the PRISMA statement (Checklist S1). A total of 49 relevant studies were identified (Figure 1). After carefully review, nine eligible case-control studies on the relationship between *XPD* Asp312Asn polymorphism and HNC risk were included in this meta-analysis [10]–[18]. Table 1 presents the main characteristics of these studies. Seven studies involved Caucasian populations [10]–[12], [14]–[16], [18], whereas two studies involved Asians [13], [17]. Diverse genotyping methods were used, including PCR-SSCP, PCR-RFLP, Taqman, Real-time PCR and SEB PCR. All studies indicated that the genotypic distribution of the controls was consistent with HWE except one [17].

**Table 1:** Characteristics of case-control studies on XPD Asp312Asn polymorphism and HNC risk included in the meta-analysis.

Frist author	Year	Racial descent	Source of controls	Case	Control	Genotype distribution						Genotying type	*P* for HWE^†^
						Case			Control				
						Asp/Asp	Asp/Asn	Asn/Asn	Asp/Asp	Asp/Asn	Asn/Asn		
Sturgis	2002	Caucasian	Hospital-based	313	313	123	165	25	142	135	36	PCR-SSCP	0.650
Matullo	2006	Caucasian	Population-based	82	1094	32	46	4	418	506	170	TaqMan	0.411
An	2007	Caucasian	Hospital-based	829	854	330	395	104	370	386	98	PCR-RFLP	0.860
Majumder	2007	Asian	Hospital-based	305	387	152	119	34	205	146	36	PCR-RFLP	0.183
Harth	2008	Caucasian	Hospital-based	311	298	113	158	40	101	145	52	Real-time PCR	0.997
Abbasi	2009	Caucasian	Population-based	246	644	93	119	34	258	304	82	Real-time PCR	0.606
Jelonek	2010	Caucasian	Hospital-based	29	58	10	14	5	14	36	8	PCR-RFLP	0.052
Ji	2010	Asian	Hospital-based	264	342	235	29	0	309	30	3	SBE PCR	0.026
Gugatschka	2011	Caucasian	Population-based	291	462	116	133	42	171	208	83	TaqMan	0.158

### Meta-analysis

The main results of this meta-analysis and the heterogeneity test are shown in Table 2. Overall, no significant relationship was observed between *XPD* Asp312Asn polymorphism and HNC risk in the total populations (for Asn/Asn vs. Asp/Asp: OR = 0.95, 95%CI = 0.80–1.13, *P* = 0.550, *P*_heterogeneity_ = 0.126; Asp/Asn vs. Asp/Asp: OR = 1.11, 95%CI = 0.99–1.24, *P* = 0.065, *P*_heterogeneity_ = 0.663; Asn/Asn+Asp/Asn vs. Asp/Asp: OR = 1.07, 95%CI = 0.97–1.19, *P* = 0.189, *P*_heterogeneity_ = 0.627; Asn/Asn vs. Asp/Asp+Asp/Asn: OR = 0.87, 95%CI = 0.68–1.10, *P* = 0.243, *P*_heterogeneity_ = 0.089). Similarly, in the succeeding analysis of HWE studies, no significant association was found between *XPD* Asp312Asn polymorphism and HNC risk(for Asn/Asn vs. Asp/Asp: OR = 0.95, 95%CI = 0.80–1.14, *P* = 0.593, *P*_heterogeneity_ = 0.120; Asp/Asn vs. Asp/Asp: OR = 1.11, 95%CI = 0.99–1.24, *P* = 0.089, *P*_heterogeneity_ = 0.586; Asn/Asn+Asp/Asn vs. Asp/Asp: OR = 1.07, 95%CI = 0.96–1.19, *P* = 0.219, *P*_heterogeneity_ = 0.528; Asn/Asn vs. Asp/Asp+Asp/Asn: OR = 0.82, 95%CI = 0.69–1.11, *P* = 0.278, *P*_heterogeneity_ = 0.082). Finally, in the stratified analysis of ethnicity and study design, we also did not find any significant association between *XPD* Asp312Asn polymorphism and HNC.

**Table 2:** Summary ORs and 95% CI of XPD Asp312Asn polymorphism and HNC risk.

	Asn/Asn vs. Asp/Asp				Asp/Asn vs. Asp/Asp				Asn/Asn+Asp/Asn vs. Asp/Asp				Asn/Asn vs. Asp/Asp+Asp/Asn			
	OR	95% CI	*P*	*P* *	OR	95% CI	*P*	*P* *	OR	95% CI	*P*	*P* *	OR	95% CI	*P*	*P* *
Total	0.95	0.80–1.13	0.550	0.126	1.11	0.99–1.24	0.065	0.663	1.07	0.97–1.19	0.189	0.627	0.87	0.68–1.10	0.243	0.089†
HWE	0.95	0.80–1.14	0.593	0.120	1.11	0.99–1.24	0.089	0.586	1.07	0.96–1.19	0.219	0.528	0.82	0.69–1.11	0.278	0.082†
Ethnicity																
Asian	1.20	0.73–2.00	0.470	0.213	1.14	0.87–1.50	0.345	0.649	1.14	0.88–1.48	0.328	0.951	1.16	0.71–1.89	0.546	0.216
Caucasian	0.92	0.76–1.11	0.366	0.121	1.11	0.98–1.25	0.110	0.468	1.06	0.94–1.19	0.317	0.430	0.83	0.64–1.08	0.165	0.085†
Design																
Hospital based	1.01	0.81–1.25	0.938	0.296	1.14	1.00–1.31	0.052	0.468	1.12	0.98–1.27	0.092	0.543	0.95	0.78–1.16	0.631	0.224
Population based	0.75	0.43–1.32	0.248	0.063†	1.04	0.85–1.28	0.684	0.692	0.98	0.81–1.19	0.865	0.611	0.73	0.42–1.28	0.271	0.045†

* Test for heterogeneity.

† Estimates for random effects model.

### Sensitivity analysis

**Figure 2:**
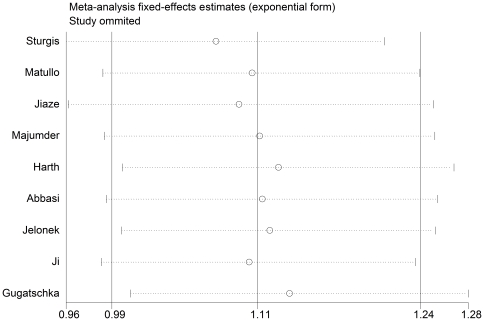
Sensitivity analysis through deletion of one study at a time to reflect the influence of the individual dataset to the pooled ORs in Asp/Asn vs. Asp/Asp model.

A single study involved in the meta-analysis was deleted each time to reflect the influence of the individual dataset to the pooled ORs. The analysis results demonstrate a borderline increased risk after excluding the studies that in Asp/Asn vs. Asp/Asp model [14], [16], [18] (Figure 2). The other corresponding pooled ORs were not materially altered (data not shown), indicating that our results are statistically robust.

### Publication bias

**Figure 3:**
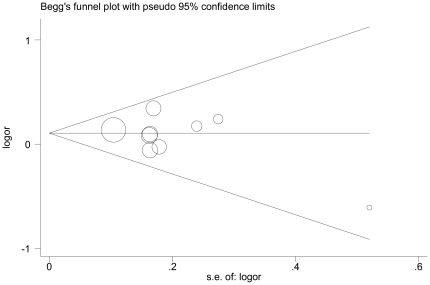
Funnel plot analysis to detect publication bias for Asp/Asn vs. Asp/Asp genotype. Each point represents a separate study for the indicated association.

Funnel plot and modified Egger's test were performed to estimate the publication bias of literature. The shapes of the funnel plots in all genetic models did not reveal any evidence of obvious asymmetry. Figure 3 shows the shapes of the funnel plots of codominant model (Asp/Asn vs. Asp/Asp), used in the studies for examining all populations. The result was further supported by analysis via modified Egger's tests. No significant publication bias was found in this meta-analysis (*P* = 0.093 for Asn/Asn vs. Asp/Asp; *P* = 0.370 for Asp/Asn vs. Asp/Asp; *P* = 0.173 for Asn/Asn+Asp/Asn vs. Asp/Asp; *P* = 0.215 for Asn/Asn vs. Asp/Asp+Asp/Asn).

## Discussion

Today, genetic susceptibility to cancer has attracted growing attention to the study of gene polymorphisms involved in tumorigenesis. The *XPD* gene has been mapped to chromosome 19q13.3 and it is composed of 23 exons. Germline mutations in the *XPD* gene can result in xeroderma pigmentosum and other diseases. The XPD protein is involved in transcription-coupled NER and is an integral member of the basal transcription factor BTF2/TFIIH complex.

The Asp to Asn change at position 312 of XPD changes the electronic configuration of amino acid and alters the interaction between XPD protein and its helicase activator [6]. Wolfe et al. demonstrated that the 312 codon polymorphisms significantly decrease the constitutive ERCC2 mRNA levels, especially in smokers [24]. Hou et al. reported that the *XPD* 312 variant allele may be associated with the reduced repair of aromatic DNA adducts [25]. Matullo et al. proposed that exposure to environmental carcinogens, such as polycyclic aromatic hydrocarbons (PAHs), also accelerate cancer development through the codon 312 variant allele of *XPD* [26].

Correlations between the polymorphisms and some cancer risks have been studied, but the results remain controversial. The *XPD* Asp312Asn polymorphism has been shown to increase the risk of bladder cancer and lung cancer, but it is not associated with breast cancer [27]–[29].

The first study, published in 2002, revealed a borderline correlation between *XPD* Asp312Asn polymorphism and HNC risk in codominant model (for Asn/Asn vs. Asp/Asp: OR, 1.41; 95% CI: 1.01–1.97) [10]. To date, no consensus has been reached on the correlation between *XPD* Asp312Asn polymorphism and HNC risk. Majumder et al. [13] found that variant genotype (Asn/Asn) at codon 312 of *XPD* is associated with increased risk of cancer among rapid and intermediate acetylators (OR = 1.9, 95% CI = 1.2–2.9). However, other studies showed that HNC risk is not significantly related to *XPD* Asp312Asn polymorphism. Ji et al. [17] found that the OR of the Asp312Asn polymorphism genotype Asp/Asn is 1.94 (95% CI = 0.92–4.08) relative to the Asp/Asp genotype. Matullo et al. [11], An et al. [12], Harth et al. [14], Abbasi et al. [15], and Jelonek et al. [16] also reported similar risks of HNC.

The present meta-analysis of nine eligible studies, including 2670 cases and 4452 controls focused on XPD Asp312Asn polymorphism and HNC risk, was performed to derive a more precise estimate of the association, but no significant association was found in the total population when all the studies were pooled. Similarly, no significant association was detected in all genetic models during the satisfied analysis based on the HWE, ethnicity and study design. Our finding is not in accordance with that previously published by Flores-Obando et al [19]. A marginally significant association was observed between the *XPD* Asp312Asn heterozygous and combined variants and HNC in their study. The considerably larger sample size of our study may account for this difference relative to the previous study.

Despite the considerable efforts to test for possible association between *XPD* Asp312Asn polymorphism and HNC risk, some limitations should be addressed. First, these results are based on unadjusted estimates that lack the original data from the eligible studies, which limits the evaluation of the effects of the gene-gene and gene-environment interactions during HNC development. Second, the sample size is still relatively small. Thus, we could not have enough statistical data to find the true relationship between *XPD* Asp312Asn polymorphism and HNC risk. Finally, each gene is known to have a moderate effect on HNC development. The combinations of certain genotypes may be more discriminating as risk factors than a single locus genotype. In our meta-analysis, linkage disequilibrium (LD) and haplotype analysis were not performed. In spite of these limitations, no publication bias was observed, and a large number of subjects still significantly guarantee the statistical power of the analysis.

In conclusion, despite these limitations,our meta-analysis suggests that *XPD* Asp312Asn polymorphism may not be associated with HNC development. In the future, large-scale case-control and population-based association studies are necessary to validate the risks identified in the present meta-analysis and to investigate the potential gene-gene and gene-environment interactions between *XPD* Asp312Asn polymorphism and HNC cancer.

## Supporting Information

Checklist S1 **PRISMA 2009 Checklist.**
(DOC)

## References

[1] Kamangar F, Dores GM, Anderson WF (2006). Patterns of cancer incidence, mortality, and prevalence across five continents: defining priorities to reduce cancer disparities in different geographic regions of the world.. J Clin Oncol.

[2] Argiris A, Karamouzis MV, Raben D, Ferris RL (2008). Head and neck cancer.. Lancet.

[3] Blot WJ, McLaughlin JK, Winn DM, Austin DF, Greenberg RS (1988). Smoking and drinking in relation to oral and pharyngeal cancer.. Cancer Res.

[4] Lichtenstein P, Holm NV, Verkasalo PK, Iliadou A, Kaprio J (2000). Environmental and heritable factors in the causation of cancer–analyses of cohorts of twins from Sweden, Denmark, and Finland.. N Engl J Med.

[5] Goode EL, Ulrich CM, Potter JD (2002). Polymorphisms in DNA repair genes and associations with cancer risk.. Cancer Epidemiol Biomarkers Prev.

[6] Coin F, Marinoni JC, Rodolfo C, Fribourg S, Pedrini AM (1998). Mutations in the XPD helicase gene result in XP and TTD phenotypes, preventing interaction between XPD and the p44 subunit of TFIIH.. Nat Genet.

[7] Winkler GS, Araujo SJ, Fiedler U, Vermeulen W, Coin F (2000). TFIIH with inactive XPD helicase functions in transcription initiation but is defective in DNA repair.. J Biol Chem.

[8] de Boer J, Hoeijmakers JH (2000). Nucleotide excision repair and human syndromes.. Carcinogenesis.

[9] Pastorelli R, Cerri A, Mezzetti M, Consonni E, Airoldi L (2002). Effect of DNA repair gene polymorphisms on BPDE-DNA adducts in human lymphocytes.. Int J Cancer.

[10] Sturgis EM, Dahlstrom KR, Spitz MR, Wei Q (2002). DNA repair gene ERCC1 and ERCC2/XPD polymorphisms and risk of squamous cell carcinoma of the head and neck.. Arch Otolaryngol Head Neck Surg.

[11] Matullo G, Dunning AM, Guarrera S, Baynes C, Polidoro S (2006). DNA repair polymorphisms and cancer risk in non-smokers in a cohort study.. Carcinogenesis.

[12] An J, Liu Z, Hu Z, Li G, Wang LE (2007). Potentially functional single nucleotide polymorphisms in the core nucleotide excision repair genes and risk of squamous cell carcinoma of the head and neck.. Cancer Epidemiol Biomarkers Prev.

[13] Majumder M, Sikdar N, Ghosh S, Roy B (2007). Polymorphisms at XPD and XRCC1 DNA repair loci and increased risk of oral leukoplakia and cancer among NAT2 slow acetylators.. Int J Cancer.

[14] Harth V, Schafer M, Abel J, Maintz L, Neuhaus T (2008). Head and neck squamous-cell cancer and its association with polymorphic enzymes of xenobiotic metabolism and repair.. J Toxicol Environ Health A.

[15] Abbasi R, Ramroth H, Becher H, Dietz A, Schmezer P (2009). Laryngeal cancer risk associated with smoking and alcohol consumption is modified by genetic polymorphisms in ERCC5, ERCC6 and RAD23B but not by polymorphisms in five other nucleotide excision repair genes.. Int J Cancer.

[16] Jelonek K, Gdowicz-Klosok A, Pietrowska M, Borkowska M, Korfanty J (2010). Association between single-nucleotide polymorphisms of selected genes involved in the response to DNA damage and risk of colon, head and neck, and breast cancers in a Polish population.. J Appl Genet.

[17] Ji YB, Tae K, Lee YS, Lee SH, Kim KR (2010). XPD Polymorphisms and Risk of Squamous Cell Carcinoma of the Head and Neck in a Korean Sample.. Clin Exp Otorhinolaryngol.

[18] Gugatschka M, Dehchamani D, Wascher TC, Friedrich G, Renner W (2011). DNA repair gene ERCC2 polymorphisms and risk of squamous cell carcinoma of the head and neck.. Exp Mol Pathol.

[19] Flores-Obando RE, Gollin SM, Ragin CC (2010). Polymorphisms in DNA damage response genes and head and neck cancer risk.. Biomarkers.

[20] Little J, Bradley L, Bray MS, Clyne M, Dorman J (2002). Reporting, appraising, and integrating data on genotype prevalence and gene-disease associations.. Am J Epidemiol.

[21] Lau J, Ioannidis JP, Schmid CH (1997). Quantitative synthesis in systematic reviews.. Ann Intern Med.

[22] DerSimonian R, Laird N (1986). Meta-analysis in clinical trials.. Control Clin Trials.

[23] Harbord RM, Egger M, Sterne JA (2006). A modified test for small-study effects in meta-analyses of controlled trials with binary endpoints.. Stat Med.

[24] Wolfe KJ, Wickliffe JK, Hill CE, Paolini M, Ammenheuser MM (2007). Single nucleotide polymorphisms of the DNA repair gene XPD/ERCC2 alter mRNA expression.. Pharmacogenet Genomics.

[25] Hou SM, Falt S, Angelini S, Yang K, Nyberg F (2002). The XPD variant alleles are associated with increased aromatic DNA adduct level and lung cancer risk.. Carcinogenesis.

[26] Matullo G, Palli D, Peluso M, Guarrera S, Carturan S (2001). XRCC1, XRCC3, XPD gene polymorphisms, smoking and (32)P-DNA adducts in a sample of healthy subjects.. Carcinogenesis.

[27] Pabalan N, Francisco-Pabalan O, Sung L, Jarjanazi H, Ozcelik H (2010). Meta-analysis of two ERCC2 (XPD) polymorphisms, Asp312Asn and Lys751Gln, in breast cancer.. Breast Cancer Res Treat.

[28] Zhang J, Qiu LX, Leaw SJ, Hu XC, Chang JH (2011). The association between XPD Asp312Asn polymorphism and lung cancer risk: a meta-analysis including 16,949 subjects.. Med Oncol.

[29] Wang F, Chang D, Hu FL, Sui H, Han B (2008). DNA repair gene XPD polymorphisms and cancer risk: a meta-analysis based on 56 case-control studies.. Cancer Epidemiol Biomarkers Prev.

